# Elements for Trial Without Catheter (TWOC) Success in Benign Prostatic Hyperplasia Patients: Lessons We Have Learned

**DOI:** 10.7759/cureus.50980

**Published:** 2023-12-23

**Authors:** Marius Ivanuta, Dragos Puia, Catalin Pricop

**Affiliations:** 1 Urology, "C.I. Parhon" Clinical Hospital, Iasi, ROU; 2 Urology, University for Medicine and Pharmacy "Grigore T. Popa", Iasi, ROU; 3 Urology, University for Medicine and Pharmacy "Grigore T. Popa", Iași, ROU

**Keywords:** dutasteride, alpha-blocker, trial without catheter, urinary retention, benign prostatic hyperplasia

## Abstract

Background: Benign prostatic hyperplasia (BPH) is a progressive disease that causes low urinary tract symptoms (LUTS). As prostatic volume grows, the prostatic urethra may become completely obstructed, resulting in full urine retention and acute hypogastric pain. Our research aimed to identify the optimal trial without catheter (TWOC) therapeutic approach and identify those factors that are associated with the recurrence of complete urinary retention (CUR).

Methodology: The study enrolled with complete urinary retention and BPH were included in the study, after the insertion of a Foley catheter. The patients received tamsulosin 0.4 mg/day as an alpha-blocker treatment. In our investigation, patients who encountered complete urinary retention were randomly categorized into four groups based on the duration of urinary catheterization as determined by the attending urologist.

Results: Maintaining the urethrovesical catheter for three to seven days was related to the highest success of spontaneous urination, which was statistically significant compared to other study groups. (p=0.0007). Age over 70 years, no alpha-blocker before the urinary retention episode, and prostatic volume exceeding 50 ml were all associated with decreased TWOC efficacy. We found the highest rates of spontaneous urination were after three to seven days of urinary catheterization.

Conclusion: BPH and complete urine retention can be managed by TWOC in many cases. Several factors affect the test's efficacy. Prolonged urinary catheter maintenance over seven days, prostatic volume over 50 ml, and age over 70 years are poor prognostic indicators.

## Introduction

Benign prostatic hyperplasia (BPH) is a progressive pathology that causes low urinary tract symptoms (LUTS). As the prostatic volume increases, there is a risk of complete obstruction of the prostatic urethra, causing complete urinary retention (CUR), characterised by the inability to initiate spontaneous urination and associated with hypogastric pain [1,2]. CUR is a urological emergency, with the immediate treatment standard being either urethrovesical catheter drainage or, in some cases, a suprapubic cystostomy. However, there is no consensus on how to manage the patient after the emergency has been solved. While some clinicians prefer to perform transurethral prostate resection after CUR installation, others perform TWOC (trial without catheter) as an intermediate step, thus avoiding unnecessary intervention. The observed correlation between emergency surgery and elevated mortality rates within 30 days, as well as a greater incidence of postoperative complications and the potential for prolonged catheterization to cause morbidity, has resulted in an increasing acceptance of the TWOC strategy [3,4]. Due to the limited availability of data and the lack of consensus in the literature, we conducted a prospective study to determine the most effective treatment strategy for successful TWOC and identify factors associated with recurrent CUR.

## Materials and methods

To achieve the objective of this research, a prospective study was conducted between June 2022 and June 2023 at the Urology and Renal Transplant Clinic of the "C.I. Parhon" Clinical Hospital, Iasi, Romania. Patients with CUR and BPH referred to our clinic, in which a urethrovesical catheter was inserted during an episode of complete urinary retention, were enrolled. Demographics, history of BPH, prostate size determined by digital rectal examination, prostatic volume by ultrasound, drained volume, the presence of ureterohydronephrosis secondary to obstruction, intravesical protrusion of the prostate (IPP), prior usage of α1-blockers, 5-α1 reductase inhibitors (5-ARI), antibiotics, and nonsteroidal anti-inflammatory drugs (NSAIDs) before TWOC was recorded. The patients received tamsulosin 0.4 mg/day as an alpha-blocker treatment.

In the present investigation, patients who encountered CUR were categorised into four groups based on the duration of urinary catheterisation as determined by the attending urologist. The patients were enrolled in order of presentation to the hospital, and the randomization was performed by the fact that each on-call urologist performed TWOC at a fixed interval, the same for all patients he examined. These groups were designated as A (3-7 days), B (7-14 days), C (14-21 days), and D (over 21 days). At the end of the indicated period, the patients returned to the outpatient clinic, at which time the urethrovesical catheter was removed. The patients were reevaluated clinically and by ultrasound within 24 hours of removing the urinary catheter, measuring the residual urinary volume. TWOC was considered successful if, within 24 hours, the patient did not require reinsertion of a urinary catheter and the bladder residue was below 100 ml during the re-evaluation. Any other situation was considered a failure of TWOC.

We excluded patients who were suspected of having urine retention due to disorders other than BPH (such as prostate cancer, pelvic trauma, acute prostatitis, urethral strictures, or neurological bladder). Patients who were suspected of having a urinary infection were also excluded.

Ethical approval for this study was obtained from the Institutional Ethics Committees of 'C.I. Parhon' Clinical Hospital (No. 3530/06.05.2022). Informed consent was obtained from all participants included in the study. All methods were carried out in accordance with relevant guidelines and regulations.

Statistical analysis was performed using SPSS software, version 27.0 (IBM Corp., Armonk, NY). Statistical significance was set at p < 0.05. Chi-squared or Fisher exact tests were used for qualitative variables, and Student's t-test was used for quantitative ones. A p-value < 0.05 was considered statistically significant.

## Results

We enrolled a total of 170 patients. Table 1 summarises the group and patient characteristics. Referring to the group's general structure, there were no statistically significant differences in origin, environment, mean age, history of BPH, prostatic volume, or severity of LUTS before the episode of urinary retention.

**Table 1:** Characteristics of the patients n: number; (%): percentage; BPH: Benign prostatic hyperplasia; LUTS: low urinary tract symptoms; CUR: complete urinary retention; IPP: intravesical prostate protrusion; SD: standard deviation. p: Pearson’s Chi-Squared, p<0.05 - statistically significant.

Parameters	Group A		Group B		Group C		Group D		p
	n	%	n	%	n	%	n	%	
Rural	32	58.18	31	67.40	28	73.68	17	54.83	0.29
Urban	23	41.81	15	32.60	10	26.31	14	45.17	
Mean age (years)	73.3		74.6		70.5		68.3		0.53
51-60 years	9	16.36	9	19.56	12	31.57	6	19.35	
61-70 years	30	54.54	28	60.86	20	52.63	17	54.83	
≥ 71 years	16	29.09	9	19.56	6	15.78	8	25.80	
History of BPH									0.01*
Yes	7	12.72	19	41.30	10	26.31	10	32.25	
No	48	87.27	27	58.69	28	73.68	21	67.74	
LUTS prior CUR									0.75*
Mild	18	32.72	15	32.60	15	39.47	14	45.16	
Moderate	20	36.36	20	43.47	14	36.84	8	26.80	
Severe	17	30.90	11	23.91	9	23.68	9	29.03	
IPP									0.28
Yes	12	21.81	16	34.78	15	39.47	9	29.03	
No	43	78.18	30	65.21	23	60.53	22	70.96	
α-blocker before CUR									0.14
Yes	28	50.90	19	41.30	14	36.84	9	29.03	
No	27	49.09	27	58.69	24	63.15	23	74.19	
Mean prostatic volume (ml)	94.6		85.5		89.2		55.5		0.233
The urinary volume before catheterisation ml (SD)	676.25 (±145.4)		670.9 (±287.4)		733.33 (±411.9)		798.8 (±304.9)		0.335

In our study, maintaining the urethrovesical catheter for three to seven days was associated with the highest success rate of TWOC, the difference being statistically significant compared to the other groups (p=0.0007), as can be observed in Table 2.

**Table 2:** Elements impacting TWOC n: number; (%): percentage; TWOC: trial without catheter, CUR: complete urinary retention; IPP: intravesical prostate protrusion; OR: odds ratio. p: Pearson’s Chi-Squared, p<0.05 - statistically significant.

Parameters	Success of TWOC		Failure of TWOC		p
	n	%	n	%	
Group A	39	70.90	16	29.10	0.0007*
Group B	17	36.95	29	63.04	
Group C	14	36.84	24	63.15	
Group D	12	38.70	19	61.29	
Age					0.0001*
51-60 years	27	64.28	15	35.71	
61-70 years	45	54.21	38	45.78	
Over 71 years	10	22.22	35	77.77	
α-blocker before CUR					0.0001* (OR =4.24)
Yes	48	68.57	22	31.42	
No	34	34	66	66	
IPP					0.10
Yes	30	57.70	22	42.30	
No	52	44.06	66	55.93	

Table 3 summarises the factors that correlate with the success of TWOC related to each age category. We observed a correlation between a reduced probability of successful TWOC and three factors: age over 70 years, lack of alpha-blocker treatment before the urinary retention episode, and prostatic volume over 50 ml. Our study indicates a significant correlation between the utilisation of NSAIDs and favourable outcomes, particularly in the 61 to 70 years age group. The positive effects of 5-ARI were exclusively observed in patients aged 70 years or older.

**Table 3:** Age-related factors and TWOC success elements TWOC: trial without catheter; n: number; (%): percentage; NSAID: nonsteroidal anti-inflammatory drugs; 5-ARI: 5-alpha-reductase inhibitors; OR: odds ratio p: Pearson’s Chi-Squared, p<0.05 - statistically significant.

Category 51-60 years	Parameters	Success of TWOC		Failure of TWOC		p
		n	%	n	%	
	TWOC					0.03*
	Group A	15	88.23	2	11.76	
	Group B	4	50	4	50	
	Group C	5	62.5	3	37.5	
	Group D	3	33.33	6	66.67	
	NSAID treatment					0.3
	Yes	10	55.55	8	44.45	
	No	17	70.83	7	29.16	
	5-ARI treatment					0.8
	Yes	3	60	2	40	
	No	24	64.86	13	35.13	
	Prostatic volume					0.04* (OR =4)
	Under 50 cmc	21	75	7	25	
	Over 50 cmc	6	42.85	8	57.14	
Category 61-70 years	TWOC					0.07*
	Group A	19	73.07	7	26.92	
	Group B	7	41.17	10	58.82	
	Group C	7	35	13	65	
	Group D	8	50	8	50	
	NSAID treatment					0.02* (OR =2.71)
	Yes	25	67.56	12	32.43	
	No	20	43.47	26	56.52	
	5-ARI treatment					0.89
	Yes	10	55.55	8	44.45	
	No	35	53.84	30	46.15	
	Prostatic volume					0.05* (OR =2.48)
	Under 50 cmc	33	62.26	20	37.73	
	Over 50 cmc	12	40	18	60	
Over 70 years	TWOC					0.27
	Group A	5	41.66	7	58.34	
	Group B	2	11.76	15	88.23	
	Group C	2	20	8	80	
	Group D	1	16.66	5	83.33	
	NSAID treatment					0.26
	Yes	22	61.11	14	38.88	
	No	23	48.93	24	51.06	
	5-ARI treatment					0.02* (OR =6)
	Yes	6	54.54	5	45.46	
	No	4	16.66	20	83.33	
	Prostatic volume					0.04* (OR =2.74)
	Under 50 ml	31	64.58	17	35.41	
	Over 50 ml	14	40	21	60	

## Discussion

The present study aimed to highlight the importance of TWOC as a valuable approach in managing urinary retention. The potential of TWOC to improve patient comfort and quality of life is one of its main benefits. Indwelling urinary catheters frequently result in pain, discomfort, and urinary tract infections, all of which have a negative effect on a patient's health. Long-term catheter use increases the risk of infections related to healthcare, such as catheter-associated urinary tract infections (CAUTIs). These infections not only strain the patients but also increase the expense of healthcare and the use of resources. Clinicians may reduce the likelihood of CAUTIs and decrease the financial burden of their treatment by using a TWOC strategy [5,6].

The data from the literature on the success of TWOC presents relatively contradictory data. Fitzpatrick et al. demonstrated in large groups of patients that the success rate of TWOC is estimated at 61.4%, which is higher than that obtained in our group of patients, for which the overall success rate of TWOC was 48.23% [2]. This difference can be explained on the one hand in the context of the numerical difference between the two studies and, on the other hand, the different strategies for maintaining the urinary catheter. Hagiwara et al. report distinct data in their clinical trial regarding the success rate of TWOC in patients with complete urinary retention who underwent maximal treatment. Their findings indicate an 88% success rate. However, in this study, the maximum duration of a urinary catheter was 12 weeks, during which multiple suppressions of the urinary catheter were performed [7]. Different data are also reported by Bansal et al., who, on a batch of over 2000 patients, report a success rate of approximately 33% [8].

Our study aimed to determine the ideal duration for urinary catheter maintenance. Based on our findings, catheter use for three to seven days is associated with the highest success rates in TWOC. In contrast to previous research conducted by Caine et al. [9], there is a negative correlation between the duration of catheter maintenance and the success of TWOC. Our results align with previous research suggesting that extended urinary catheter use does not lead to better outcomes [1].

The probability of successful TWOC seems to be correlated both with anthropometric parameters and with the volumetric characteristics of the prostate. Prior studies have demonstrated that the success rate of TWOC in elderly patients is comparatively lower than in younger patients. According to Bansal et al., it was observed that the age of patients who underwent successful TWOC was significantly lower than those who did not (62.7 vs. 67.7 years) [8]. This relationship was also observed by Ko et al., who reported that successful TWOC was more common in patients with a lower mean age compared to those who experienced urinary retention following catheter removal (60 years vs. 66 years [10]. Our study's data are comparable with previous studies, indicating a correlation between age and decreased success rates of TWOC. The success rates observed in our study were 64.28% for individuals aged 51-60 years, 54.21% for individuals aged 61-70 years, and 22.22% for individuals aged over 70 years.

As detected by ultrasound, IPP may result in a valve effect on the bladder neck. Some studies suggest that the degree of obstruction increases with the severity of the protrusion. According to the findings of Bhomi et al., patients with a higher IPP have a lower probability of successful TWOC [11]. Tan and Foo have reported on the failure rate of voiding trials about IPP grade. The study found that the failure rate was 36% in grade 1, 58% in grade 2, and 67% in grade 3, which was statistically significant [12]. Similarly, Bansal's research found that patients with a successful TWOC had a smaller intravesical protrusion than those with a failed TWOC [8]. In contrast to the data from the literature, in our study, IPP did not constitute a statistically significant factor in the success of TWOC.

The prostatic volume was a significant parameter in our research regarding the likelihood of resuming micturition after catheter suppression. Regardless of age, patients with prostate volumes less than 50 ml demonstrated increased likelihoods of spontaneous urination resumption (age category 51-60 years, odds ratio (OR)=4; age category 61-70 years, OR=2.48; age category over 70 years, OR=2.72). Prior research conducted by Kumar et al. [13] and Mahadik et al. [14] has indicated that a larger prostate size is correlated with failure TWOC. According to Bhomi and Bhattachan's study, patients who underwent successful TWOC had a notably lower prostate volume compared to those who experienced unsuccessful TWOC (46 vs. 68.4 grams) [11].

Our research aimed to determine the impact of medication on the success rate of TWOC. The findings revealed that patients who were previously diagnosed with BPH and were undergoing alpha-blocker treatment had a greater likelihood of resuming spontaneous micturition following an episode of complete urinary retention (68.57%). In contrast, patients who initiated alpha-blocker treatment only after the occurrence of urine retention had a lower probability of success (31.42%). An extensive amount of research supports the importance of alpha-blocker therapy in the context of TWOC. McNeill et al. demonstrated that administering alpha-blocker medication led to a higher rate of successful TWOC among all participants (61.9% versus 47.9%) [15].

The value of alpha-blocker treatment in BPH is well established, but the importance of other drug classes in the success of TWOC has not been reported to our knowledge. Consequently, we assessed the role of nonsteroidal anti-inflammatories and 5-ARI medication among age groups.

Recent histological investigations have revealed that intraprostatic inflammatory infiltration is observed in 43-98% of BPH tissues. Consequently, the administration of NSAID drugs appears to be a reasonable approach to managing this disease. According to a systematic review, there is evidence to suggest that NSAIDs can lead to an improvement in symptoms and urinary flow that are associated with BPH [16]. Our study indicated that the administration of NSAIDs improved the success rate of TWOC in patients aged 61 to 70 years as compared to those who did not receive this treatment. The statistical analysis revealed no significant correlation between the age groups of 51-60 years and over 71 years.

Regarding another medication frequently used in BPH, 5-ARI, several studies have demonstrated that in addition to the effect on prostatic volumetry, this therapeutic class also proves its effectiveness by reducing the microvascular density of the prostatic parenchyma, thus resulting in a decrease in bleeding intraoperatively during transurethral prostate resection as well as postoperatively, significantly reducing the probability of macroscopic haematuria [17]. No research has been conducted on the utility of 5-ARI in TWOC. Our data showed that patients who received dutasteride treatment had a greater likelihood of spontaneous urination (OR = 6), but this was only observed in patients over 70 years old.

Our research has a series of limitations, one of which is represented by the relatively small sample. Another aspect is related to the fact that the follow-up period of the patients was reduced. For this reason, at this moment, we cannot estimate to what extent the recurrence of urinary retention occurred in some patients.

## Conclusions

TWOC is a feasible alternative for the treatment of individuals with benign prostatic hyperplasia and complete urinary retention. However, several factors impact the efficacy of this therapeutic approach. Prolonged urinary catheters exceeding seven days, prostatic volume exceeding 50 ml, and age over 70 years are considered unfavorable prognostic factors. Initiation of alpha-blockers before urinary retention, along with the addition of an NSAID in patients aged 61-70 years and a 5-ARI in patients over 70 years, is associated with increased success rates of TWOC.
